# Supplementary material for: Shift in VEGFA isoform balance towards more angiogenic variants is associated with tumor stage and differentiation of human hepatocellular carcinoma
Source: PeerJ. 2018 Jun 5;6:e4915. doi: 10.7717/peerj.4915 (PMC5993022; doi:10.7717/peerj.4915)
Supplement: Supplemental Information 1 [file peerj-06-4915-s001.docx]

**Supplemental Table S1.** List of primers used in RT-PCR and RT-qPCR analysis.

| Primer name | Forward primer | Reverse primer | T_ann,_ °C^a^ |
| --- | --- | --- | --- |
| TBP | TGCACAGGAGCCAAGAGTGA | ACTTCACATCACAGCTCCCCA | 62.8 |
| VEGFA-iso | TGCGGATCAAACCTCACCAA | CCTCCGGACCCAAAGTGCT | 68.0 |
| VEGFA-total | CCCACTGAGGAGTCCAACATC^b^ | CTGCATTCACATTTGTTGTGCTG | 62.2 |
| VEGFA-intron5 | CCCACTGAGGAGTCCAACATC^b^ | CCCCAACAGAGGTAGCCAAG | 62.2 |
| VEGFA-xxxb | ACGTACTTGCAGATCTCTCACCA | CCTCCGGACCCAAAGTGCT | 68.6 |
| VEGFA-189 | CCCACTGAGGAGTCCAACATC^b^ | AAGGCCCACAGGGAACGC | 62.2 |
| VEGFA-165 | CCCACTGAGGAGTCCAACATC^b^ | AAGGCCCACAGGGATTTTCTT | 62.2 |
| VEGFA-121 | CCCACTGAGGAGTCCAACATC^b^ | TCGGCTTGTCACATTTTTCTTGT | 62.2 |

^a^T_ann_ – annealing temperature for each pair of primers was experimentally optimized to minimize the non-specific amplification without reducing PCR efficiency.

^b^Common forward primer in combination with different transcript-specific reverse primers were used to detect different isoforms and transcript variants of VEGFA mRNA.
