# Supplementary material for: Shift in VEGFA isoform balance towards more angiogenic variants is associated with tumor stage and differentiation of human hepatocellular carcinoma
Source: PeerJ. 2018 Jun 5;6:e4915. doi: 10.7717/peerj.4915 (PMC5993022; doi:10.7717/peerj.4915)
Supplement: Supplemental Information 2 — “Unique sequence regions” represent fragments of PCR products identified by direct sequencing (Data S2) that were long enough to be unambiguously matched to certain VEGFA transcripts or groups of transcripts. [file peerj-06-4915-s002.docx]

**Supplemental Table S2.** Correspondence between PCR products and target VEGFA transcripts. “Unique sequence regions” represent fragments of PCR products identified by direct sequencing (Data S2) that were long enough to be unambiguously matched to certain VEGFA transcripts or groups of transcripts.

| PCR product | Unique sequence region | Corresponding NCBI Nucleotide transcript |
| --- | --- | --- |
| VEGFA-iso (523 bp) | CGGTATAAGTCCCGGAGCGTTCCCTGTGGCCCTTGCTCAGAGCGGAGAAAGCATTTGTTTGTACAAGATCCGCAGACGTGTAAATGTTCCTGCAAAAACACAGACTCGCGTTGCAAGGCGAGGCAGCTTGAGTTAAACGAACGTACTTGCAGATGTGACAAGCCGAGGCGGTGAGCCGGGCAGGAGGAAGGAGCCTCCCTCAGGGTTTCGGGAACCAGATCTCTCACC | <NM_001171624.1>  (VEGFA-189) |
| VEGFA-iso (451 bp) | GATAGAGCAAGACAAGAAAATCCCTGTGGCCCTCTGCTCAGAGCGGAGAAAGCATTTGTTTGTACAAGATCCGCAGACGTGTAAATGTTCCTGCAAAAACACAGACTCGCGTTGCAAGGCGAGGCAGCTTGAGTTAAACGAACGTACTTGCAGATGTGACAAGCCGAGGCGGT | <NM_001025368.2>  (VEGFA-165) |
| VEGFA-iso (319 bp) | GATAGAGCAAGACAAGAAAAATGTGACAAGCCGAGGCGGTGAGCCGGGCAGGAGGAAGGAGCCTCCCTCAGGGTTTCGGGAACCAGATCTCTCACCAGGAAAGACT | <NM_001025370.2> (VEGFA-121) |
| VEGFA-total | CCCACTGAGGAGTCCAACATCACCATGCAGATTATGCGGATCAAACCTCACCAAGGCCAGCACATAGGAGAGATGAGCTTCCTACAGCACAACAAATGTGAATGCAG | [NM_001025366.2](http://www.ncbi.nlm.nih.gov/nuccore/NM_001025366.2)  (VEGFA-206)^a^  [NM_003376.5](http://www.ncbi.nlm.nih.gov/nuccore/NM_003376.5)  (VEGFA-189)  [NM_001025367.2](http://www.ncbi.nlm.nih.gov/nuccore/NM_001025367.2)  (VEGFA-183)^a^  [NM_001025368.2](http://www.ncbi.nlm.nih.gov/nuccore/NM_001025368.2)  (VEGFA-165)  [NM_001033756.2](http://www.ncbi.nlm.nih.gov/nuccore/NM_001033756.2)  (VEGFA-165b)^a^  [NM_001025369.2](http://www.ncbi.nlm.nih.gov/nuccore/NM_001025369.2)  (VEGFA-148)^a^  [NM_001204384.1](http://www.ncbi.nlm.nih.gov/nuccore/NM_001204384.1)  (VEGFA-145)^a^  [NM_001025370.2](http://www.ncbi.nlm.nih.gov/nuccore/NM_001025370.2)  (VEGFA-121)  [NM_001171622.1](https://www.ncbi.nlm.nih.gov/nucleotide/284172456?report=genbank&log$=nuclalign&blast_rank=3&RID=TBX5CZ4C01R)  (VEGFA-111)^a^ |
| PCR product | Unique sequence region | Corresponding NCBI Nucleotide transcript |
| VEGFA-intron5 | CCCACTGAGGAGTCCAACATCACCATGCAGATTATGCGGATCAAACCTCACCAAGGCCAGCACATAGGAGAGATGAGCTTCCTACAGCACAACAAATGTGAATGCAGACCAAAGAAAGATAGAGCAAGACAAGAAAA**GTAAGTGGCCCTGACTTTAGCACTTCTCCCTCTCCATGGCCGGTTGTCTTGGTTTGGGGCTCTTGGCTACCTCTGTTGGGG** | Normal text – mature VEGFA transcripts:  [NM_001025366.2](http://www.ncbi.nlm.nih.gov/nuccore/NM_001025366.2)  (VEGFA-206)^a^  [NM_003376.5](http://www.ncbi.nlm.nih.gov/nuccore/NM_003376.5)  (VEGFA-189)  [NM_001025367.2](http://www.ncbi.nlm.nih.gov/nuccore/NM_001025367.2)  (VEGFA-183)^a^  [NM_001025368.2](http://www.ncbi.nlm.nih.gov/nuccore/NM_001025368.2)  (VEGFA-165)  [NM_001033756.2](http://www.ncbi.nlm.nih.gov/nuccore/NM_001033756.2)  (VEGFA-165b)^a^  [NM_001025369.2](http://www.ncbi.nlm.nih.gov/nuccore/NM_001025369.2)  (VEGFA-148)^a^  [NM_001204384.1](http://www.ncbi.nlm.nih.gov/nuccore/NM_001204384.1)  (VEGFA-145)^a^  [NM_001025370.2](http://www.ncbi.nlm.nih.gov/nuccore/NM_001025370.2)  (VEGFA-121)  **Bold underlined text – genomic sequence of VEGFA intron5:**  [NC_000006.12](https://www.ncbi.nlm.nih.gov/nucleotide/568815592?report=genbank&log$=nuclalign&blast_rank=1&RID=TBYBG19801R)  *VEGFA* gene,  intron 5,  position 43778919-43778999 |
| VEGFA-xxxb | ACGTACTTGCAGATCTCTCACCAGGAAAGACTGATACAGAACGATCGATACAGAAACCACGCTGCCGCCACCACACCATCACCATCGACAGAACAGT | [NM_001033756.2](http://www.ncbi.nlm.nih.gov/nuccore/NM_001033756.2)  (VEGFA-165b)^a^ |
|  |  |  |
|  |  |  |
| PCR product | Unique sequence region | Corresponding NCBI Nucleotide transcript |
| VEGFA-189 | CCCACTGAGGAGTCCAACATCACCATGCAGATTATGCGGATCAAACCTCACCAAGGCCAGCACATAGGAGAGATGAGCTTCCTACAGCACAACAAATGTGAATGCAGACCAAAGAAAGATAGAGCAAGACAAGAAAAAAAATCAGTTCGAGGAAAGGGAAAGGGGCAAAAACGAAAGCGCAAGAAATCCCGGTATAAGTCCTGGAGCGTTCCCTGTGGGCCTT | <NM_001171624.1>  (VEGFA-189) |
| VEGFA-165 | CCCACTGAGGAGTCCAACATCACCATGCAGATTATGCGGATCAAACCTCACCAAGGCCAGCACATAGGAGAGATGAGCTTCCTACAGCACAACAAATGTGAATGCAGACCAAAGAAAGATAGAGCAAGACAAGAAAATCCCTGTGGGCCTT | <NM_001025368.2>  (VEGFA-165)  [NM_001033756.2](http://www.ncbi.nlm.nih.gov/nuccore/NM_001033756.2)  (VEGFA-165b)^a^  [NM_001025369.2](http://www.ncbi.nlm.nih.gov/nuccore/NM_001025369.2)  (VEGFA-148)^a^ |
| VEGFA-121 | CCCACTGAGGAGTCCAACATCACCATGCAGATTATGCGGATCAAACCTCACCAAGGCCAGCACATAGGAGAGATGAGCTTCCTACAGCACAACAAATGTGAATGCAGACCAAAGAAAGATAGAGCAAGACAAGAAAAATGTGACAAGCCGA | <NM_001025370.2> (VEGFA-121) |

^a^Expression of these transcript variants was not detected in NT or HCC tissue using VEGFA-iso primers.
