# Supplementary material for: Shift in VEGFA isoform balance towards more angiogenic variants is associated with tumor stage and differentiation of human hepatocellular carcinoma
Source: PeerJ. 2018 Jun 5;6:e4915. doi: 10.7717/peerj.4915 (PMC5993022; doi:10.7717/peerj.4915)
Supplement: Supplemental Information 3 [file peerj-06-4915-s003.docx]

**Supplemental Table S3.** Encoding of HCC clinicopathological features into numbers for carrying out Spearman’s correlation test.

| Characteristic | Common notation | Encoded digital notation |
| --- | --- | --- |
| Age | years | years |
| Gender | male  female | 1  2 |
| TNM staging | I  II  III  IV | 1  2  3  4 |
| BCLC staging | A  B  C  D | 1  2  3  4 |
| Tumor size | cm | cm |
| Intrahepatic metastases | no  yes | 0  1 |
| Lymph node metastases | no  yes | 0  1 |
| Distant metastases | no  yes | 0  1 |
| Tumor capsule presence | absent  feeble  prominent | 0  1  2 |
| Invasion into blood vessels | no  yes | 0  1 |
| Tumor vascularity | low  moderate  high | 1  2  3 |
| Histological differentiation,  Edmondson-Steiner grade | G1 - high  G2 - moderate  G3 - poor  G4 – undifferentiated  Gx – not applicable | 1  2  3  4  N/A |
| Alpha-fetoprotein serum level | low (<50 ng/ml)  high (>50 ng/ml) | 1  2 |
| Ascites | no  yes | 0  1 |
| Cirrhosis | no  yes | 0  1 |
| Tumor necrosis | no  yes | 0  1 |
