# Supplementary material for: Shift in VEGFA isoform balance towards more angiogenic variants is associated with tumor stage and differentiation of human hepatocellular carcinoma
Source: PeerJ. 2018 Jun 5;6:e4915. doi: 10.7717/peerj.4915 (PMC5993022; doi:10.7717/peerj.4915)
Supplement: Supplemental Information 4 [file peerj-06-4915-s004.docx]

**Supplemental Data S1.** Conditions of semi-quantitative and quantitative RT-PCR analysis.

Reverse transcription was performed using 3 μg of total RNA with 0.1 μg of random hexanucleotides and 50 units of RevertAid Reverse Transcriptase (Thermo Fisher Scientific, USA).

For semi-quantitative RT-PCR analysis 25 μl of reaction mixture contained 5 μl of cDNA template, Taq-buffer for PCR, 50 ng of each primer, 0.05 mM dNTPs and 1.25 units of TaqDNA polymerase (Sileks, Russian Federation). 30 cycles of amplification (50 sec at 95°C, 50 sec at selected annealing temperature (see Table S1), 50 sec at 72°C) were performed using T3 Thermocycler (Biometra, Germany). PCR products were analyzed by electrophoresis in 3.5% agarose gel.

For quantitative RT-PCR analysis 25 μl of reaction mixture contained 10 μl of ten-times diluted cDNA template, Taq-buffer containing SYBRI dye, 10 pM of each primer, 0.25 mM dNTPs and 1.25 units of SynTaq DNA polymerase (Syntol, Russian Federation). 45 cycles of amplification (30 sec at 95°C, 30 sec at annealing temperature (Table S1), 30 sec at 72°C) were performed using iQ5 thermal cycler (Bio-Rad, USA). Reaction specificity was checked by melt curve analysis and agarose electrophoresis.

In each experiment, three standard dilutions of samples with known copy number of corresponding PCR amplicon were processed together with experimental samples to check reaction efficiency and perform absolute quantification of VEGFA mRNA amount in examined samples. Standard samples for genes of interest were generated by cloning corresponding PCR fragments into pAL2-T plasmid vectors using Quick-TA kit (Evrogen, Russian Federation). Plasmid concentrations were estimated using Qubit 2.0 Fluorometer (Thermo Fisher Scientific, USA). Plasmid copy numbers per μl were calculated based on concentration and plasmid molar masses and additionally checked by qPCR.

Reaction efficiency in each reaction was within 98%-102%. This approach allowed us to estimate the absolute quantity of different VEGFA transcripts in each sample and calculate their fractions in total pool of all mature VEGFA transcripts. All copy number values obtained for VEGFA transcripts were then normalized to copy number value obtained for *TBP* gene used as a calibrator housekeeping gene, and these normalized values were used to calculate expression level changes in pairs of NT and HCC samples.
