# Supplementary material for: Shift in VEGFA isoform balance towards more angiogenic variants is associated with tumor stage and differentiation of human hepatocellular carcinoma
Source: PeerJ. 2018 Jun 5;6:e4915. doi: 10.7717/peerj.4915 (PMC5993022; doi:10.7717/peerj.4915)
Supplement: Supplemental Information 5 [file peerj-06-4915-s005.docx]

**Supplemental Data S2.** Verification of correspondence between PCR products and individual VEGFA isoforms by sequencing.

PCR products were separated in 3.5% agarose gel, each band was excised and extracted using PureLink Quick Gel Extraction Kit (Thermo Fisher Scientific, USA). PCR fragments generated with VEGFA-iso primers were directly sequenced using VEGFA-iso primers. Purified PCR products generated with VEGFA-189, VEGFA-165, VEGFA-121, VEGFA-total, VEGFA-intron5 and VEGFA-xxxb primers were cloned to pAL2-T plasmid vectors using Quick-TA kit (Evrogen, Russian Federation). Plasmids containing PCR amplicons were directly sequenced with standard M13 primers using ABI 3500 Genetic Analyzer and BigDye Terminator v3.1 Cycle Sequencing Kit (Applied Biosystems, USA). Sequence data confirmed that three major bands amplified by VEGFA-iso primers correspond to VEGFA-189, VEGFA-165 and VEGFA-121 isoforms (see Table S2). The fourth product, visible in some samples as a band slightly shorter than VEGFA-165 band, turned out to be a heteroduplex of VEGFA-165 and VEGFA-121 PCR products (Fig. S1). PCR products amplified by VEGFA-total, VEGFA-intron5, VEGFA-xxxb, VEGFA-189, VEGFA-165 and VEGFA-121 primers were also confirmed by sequencing to match related transcripts or groups of transcripts (Table S2).
