# Supplementary material for: Shift in VEGFA isoform balance towards more angiogenic variants is associated with tumor stage and differentiation of human hepatocellular carcinoma
Source: PeerJ. 2018 Jun 5;6:e4915. doi: 10.7717/peerj.4915 (PMC5993022; doi:10.7717/peerj.4915)
Supplement: Supplemental Information 6 [file peerj-06-4915-s006.docx]

**Supplemental Data S3.** Estimation of quantity of mature VEGFA transcripts lacking residual intron sequences.

*VEGFA* gene consists of 8 exons and 7 introns. During the maturation of full-length VEGFA pre-mRNA most of the introns are excised from the sequence and a number of VEGFA isoforms containing different sets of exons arise due to alternative splicing. However, according to our data, considerable amount of VEGFA transcripts detected in liver and HCC tissue may retain the sequences of intron 3, intron 5 or both. We did not detect any presence of other VEGFA introns in mRNA of examined tissue samples.

Possible functions of VEGFA transcripts retaining intron 3 or intron 5 are currently unknown since these transcripts do not correspond to any described protein. So it is important to consider these unspliced transcripts when evaluating the expression level of VEGFA isoforms in liver and HCC tissue.

We designed VEGFA-total, VEGFA-189, VEGFA-165 and VEGFA-121 primers in such way that they span intron 3 (Fig. 1A), thus preventing intron 3-containig transcripts from being amplified under optimized PCR conditions and affecting RT-qPCR results. VEGFA-189, VEGFA-165 and VEGFA-121 primers also span intron 5, so they only detect fully processed VEGFA transcripts. However, intron 5 is situated between conservative and variable regions of VEGFA mRNA (Fig. 1A), so it is impossible to design primers that simultaneously amplify all VEGFA variants and span intron 5. To overcome this problem, we designed VEGFA-intron5 primers (Table S1) that anneal at sites inside exon 3 and intron 5 and detect only transcripts lacking intron 3 but containing intron 5 (Table S2). We then subtracted the amount of transcripts retaining intron 5 from the amount of VEGFA transcripts estimated using VEGFA-total primers (that lack intron 3 but may contain intron 5), thus calculating the quantity of all fully processed mature VEGFA transcripts.

VEGFA-xxxb primers do not span intron 3 or intron 5 and do not discern mature VEGFA mRNA from unspliced transcripts. However, since VEGFA-xxxb fraction in total VEGFA mRNA pool is close to negligible, it does not significantly affect our subsequent calculations.
