# Supplementary figures and images for: Shift in VEGFA isoform balance towards more angiogenic variants is associated with tumor stage and differentiation of human hepatocellular carcinoma
Source: PeerJ. 2018 Jun 5;6:e4915. doi: 10.7717/peerj.4915 (PMC5993022; doi:10.7717/peerj.4915)

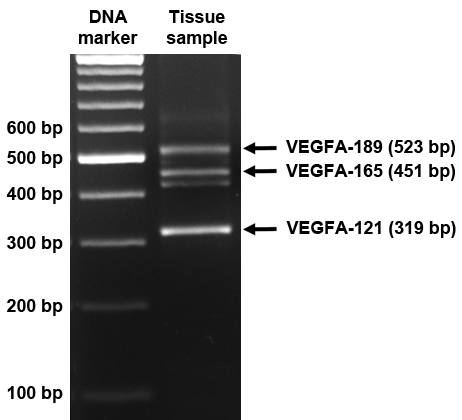

Supplement: Supplemental Information 8 — Lengths of DNA markers are indicated on the left, VEGFA isoforms are indicated on the right. The unmarked band under VEGFA-165 PCR product corresponds to heteroduplex of VEGFA-165 and VEGFA-121 PCR products but not to any certain VEGFA isoform, according to sequencing data (Supplemental Data S2). [file peerj-06-4915-s008.png]

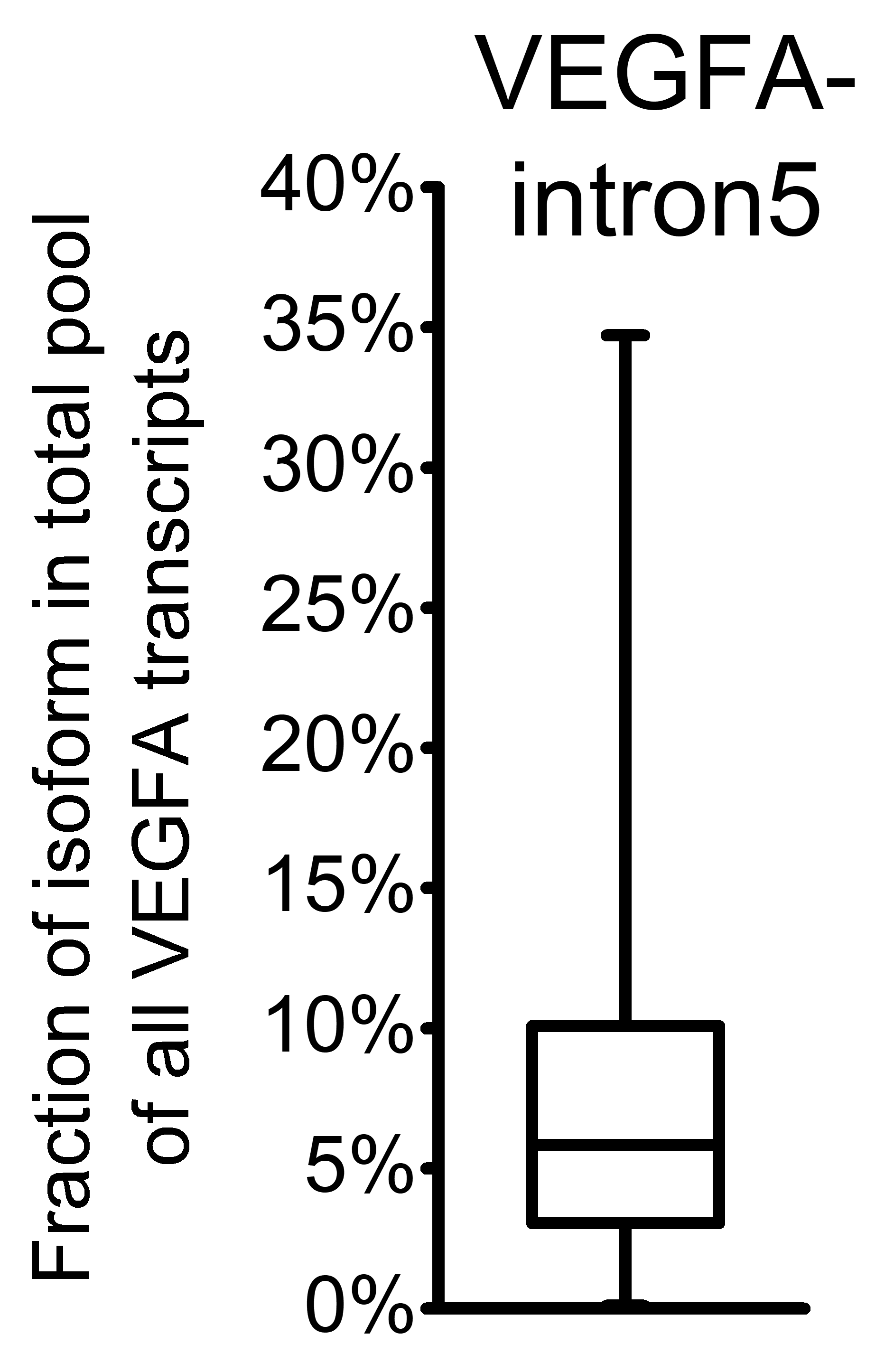

Supplement: Supplemental Information 9 — Data for all samples set (n=100) are presented as box-and-whisker plot. [file peerj-06-4915-s009.png]

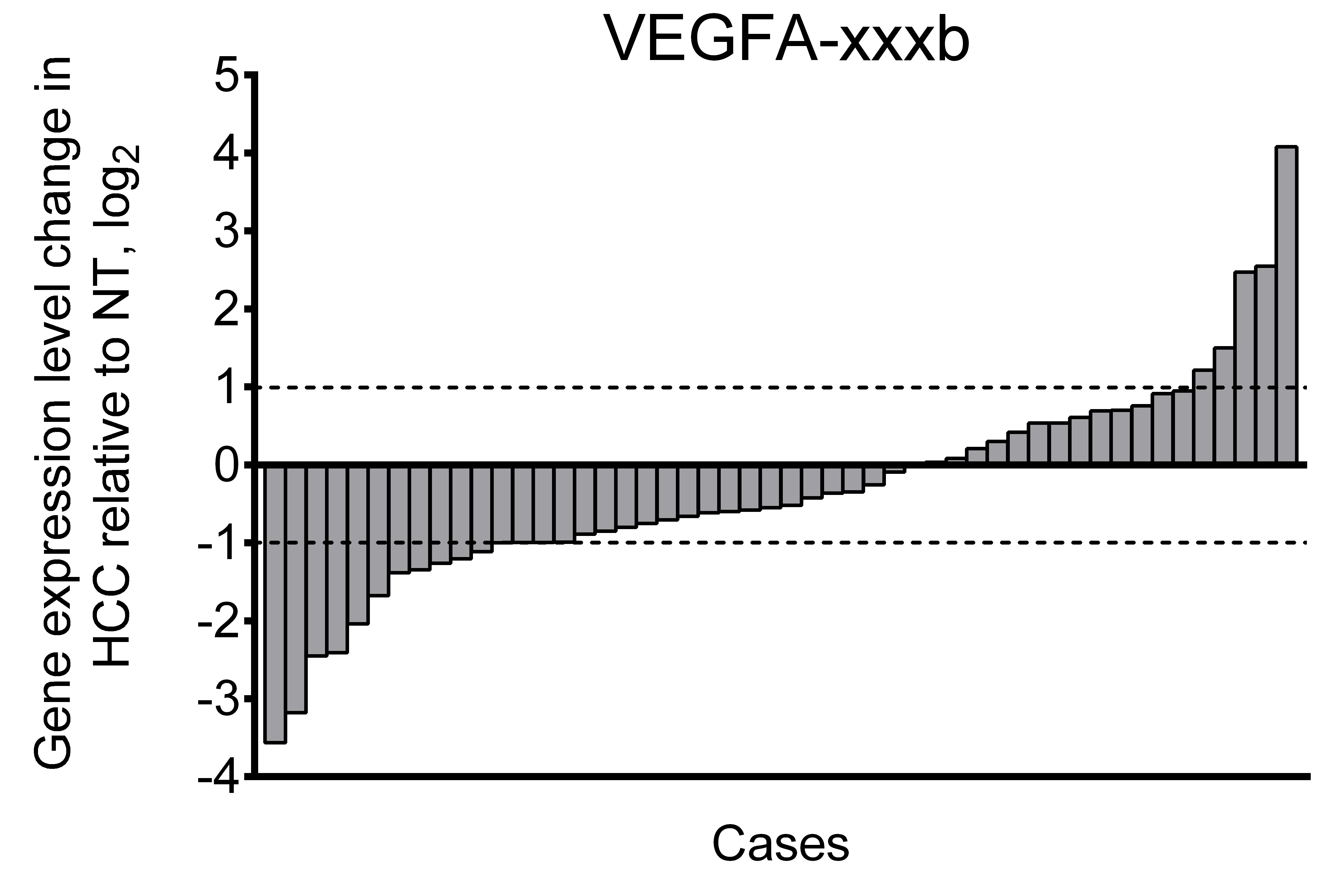

Supplement: Supplemental Information 10 — Data are presented as NT/HCC ratios in logarithmic scale. [file peerj-06-4915-s010.png]
